# The effects of intravenous tramadol vs. intravenous ketamine in the prevention of shivering during spinal anesthesia: A meta-analysis of randomized controlled trials

**DOI:** 10.3389/fmed.2022.1011953

**Published:** 2022-12-05

**Authors:** Efrem Fenta, Simegnew Kibret, Metages Hunie, Tadese Tamire, Yewlsew Fentie, Shimelis Seid, Diriba Teshome

**Affiliations:** Department of Anesthesia, College of Health Sciences, Debre Tabor University, Debre Tabor, Ethiopia

**Keywords:** ketamine, tramadol, spinal, anesthesia, shivering

## Abstract

**Background:**

Shivering is a common complication after subarachnoid administration of local anesthetics. Intravenous ketamine and tramadol are widely available anti-shivering drugs, especially in developing settings. This meta-analysis aimed to compare the effects of intravenous ketamine vs. tramadol for post-spinal anesthesia shivering.

**Materials and methods:**

PubMed/MEDLINE, Web of Science, Cochrane Library, Embase, and Google Scholar databases were used to search for relevant articles for this study. Mean difference (MD) with 95% confidence interval (CI) was used to analyze continuous outcomes, and risk ratio (RR) with 95% CI to analyze categorical results. The heterogeneity of the included studies was assessed using the I2 test. We utilized Review Manager 5.4.1 to perform statistical analysis.

**Results:**

Thirteen studies involving 1,532 patients were included in this meta-analysis. Ketamine had comparable effects in preventing post-spinal anesthetics shivering [RR = 1.06; 95% CI (0.94, 1.20), *P* = 0.33, *I*^2^ = 77], and onset of shivering [MD = −0.10; 95%CI (– 2.68, 2.48), *P* = 0.94, *I*^2^ = 0%], lower incidences of nausea and vomiting [RR = 0.51; 95%CI (0.26, 0.99), *P* = 0.05, *I*^2^ = 67%], and lower incidences of bradycardia [RR = 0.16; 95%CI (0.05, 0.47), *P* = 0.001, *I*^2^ = 33%], higher incidence of hallucinations [RR = 12; 95%CI (1.58, 91.40), *P* = 0.02, *I*^2^ = 0%], and comparable effects regarding the incidences of hypotension [RR = 0.60; 95%CI (0.30, 1.21), *P* = 0.15, *I*^2^ = 54%] as compared to tramadol.

**Conclusions:**

Intravenous ketamine and tramadol are comparable in the prevention of post-spinal anesthetic shivering. Ketamine had a better outcome with less occurrences of nausea, vomiting, and bradycardia. However, ketamine was associated with higher incidences of hallucinations than tramadol.

## Introduction

Shivering is defined as an involuntary, repetitive activity of skeletal muscles to raise the core body temperature (1–5). Spinal anesthesia is known to decrease the shivering threshold, preceded by core hypothermia and vasoconstriction above the level of the block (6). The review of 21 studies reported that the median incidence of shivering related to neuraxial anesthesia was 55% in ranges of 40% to 64% (7).

Shivering may have beneficial thermoregulatory effects; however, it is a distressing experience and causes several undesirable detrimental effects (8). It leads to an increase in oxygen consumption and carbon dioxide production, intraocular and intracranial pressure (9–11). It may also lead to an increase in sympathetic tone that enhances the chances of myocardial ischemia (12, 13), pain (14), and bleeding (15). Shivering may impede monitoring techniques (non-invasive blood pressure, electrocardiogram, and pulse oximetry) (16–19).

A variety of pharmacologic and non-pharmacologic techniques for the prevention and treatment of shivering have been used; however, there is no globally accepted preferred technique for the treatment or prevention of post-spinal anesthetic shivering (7). Ketamine acts as a competitive N-methyl-D-aspartic acid receptor antagonist and can control post-spinal anesthetic shivering; In addition, it may decrease core-to-peripheral redistribution of heat by direct central sympathetic stimulation and by blocking inhibition of norepinephrine uptake into postganglionic sympathetic nerve endings, and it has a κ-opioid agonist property (17, 20–23). Tramadol has a μ-opioid agonist effect with minimum effect at kappa and delta receptors. Tramadol inhibits the re-uptake of serotonin and norepinephrine at the spinal cord level, which increases 5-hydroxytryptamine production. These actions of the drug make it effective in preventing and controlling post-spinal anesthetic shivering (24–27).

Intravenous tramadol and ketamine are widely available and cheap drugs, especially in the low resource settings. However, there is no high-quality data (meta-analysis) or large-sized randomized controlled trials on the relative efficacy and safety (anti-shivering agent with lesser side effects) of intravenous ketamine vs. tramadol. Hence, this meta-analysis aimed to compare the effects of intravenous tramadol vs. ketamine in preventing shivering after spinal anesthesia and associated side effects.

## Materials and methods

This study is reported as per Preferred Reporting Items for Systematic and Meta-analysis. Thirteen randomized controlled trials with a total of 1,532 patients were included. This meta-analysis was registered in Prospero with registration number *CRD42022342030* on July 5, 2022.

### Search strategy

PubMed/MEDLINE, Web of Science, Cochrane Library, Embase, and Google Scholar databases were used for searching relevant articles. The terms used for searching were “Ketamine,” “Tramadol,” “Spinal Anesthesia,” and “Shivering” through June 2022.

### Inclusion criteria

Patients undergoing surgery under spinal anesthesia; studies that compare intravenous ketamine with intravenous tramadol on shivering; the incidence of side effects reported in both tramadol and ketamine groups; and randomized controlled trials were included.

### Data extraction

The titles and abstracts of all articles were reviewed by two authors. Studies that are deemed to fall outside the inclusion criteria were excluded. Full paper copies of the remaining studies were reviewed by two authors (EF and DT) independently, and decisions made regarding selection/rejection. Any disagreements arising were resolved by a third reviewer (TT). The authors' name, publication year, characteristics of study participants, sample size, type of surgery, the dose and type of drug used for spinal anesthesia, the anti-shivering dose of intravenous ketamine and intravenous tramadol, and the outcomes of each included study were extracted.

### Evaluation of the risk of bias (quality) assessment

The risk of bias was assessed using the Cochrane risk of bias tool and graded as low, unclear, or high risk of bias by two researchers independently. The included articles were rated according to random sequence generation (selection bias), allocation concealment (selection bias), blinding of participants and personnel (performance bias), blinding of outcome assessment (detection bias), incomplete outcome data (attrition bias), selective reporting (reporting bias), and other bias. The disagreements between the researchers arising were resolved by a third reviewer.

### Statistical analysis

We performed a meta-analysis of the effects of intravenous tramadol vs. ketamine in preventing post-spinal anesthetic shivering.

**Figure 1:**
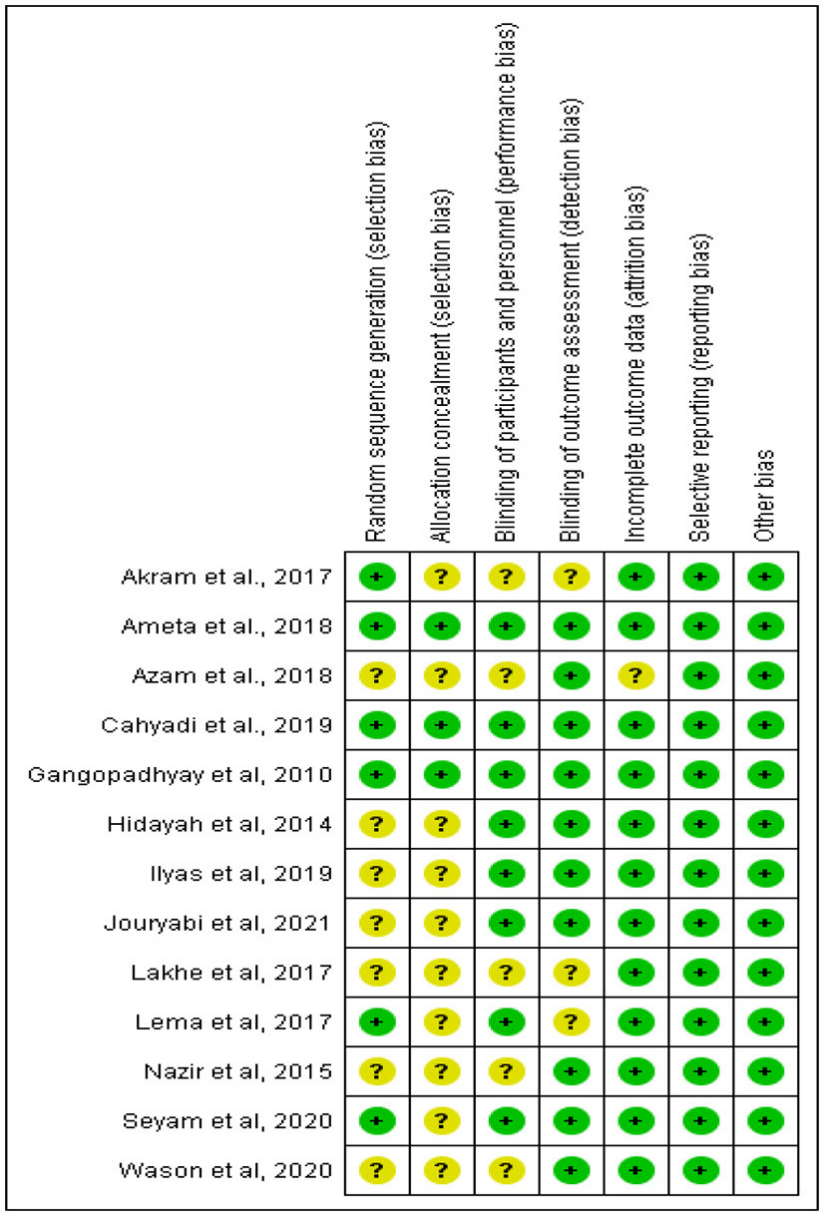
The risk of bias assessment of included studies.

The Review Manager 5.4.1 (Cochrane Library, Oxford, UK) was used for this meta-analysis (Figure 1). The effective rate of shivering, the incidence rate of nausea and vomiting, hypotension, bradycardia, and hallucination were expressed in risk ratio (RR) with a 95% confidence interval (CI); and the onset of shivering in minutes was expressed in mean difference (MD) with 95% confidence interval (CI). If the *I*^2^ was >50% or <50%, a fixed-effect model and a random-effect model, respectively, were utilized. The symmetry of the funnel plot showed that there was no publication bias.

## Results

### Characteristics of the included studies

**Figure 2:**
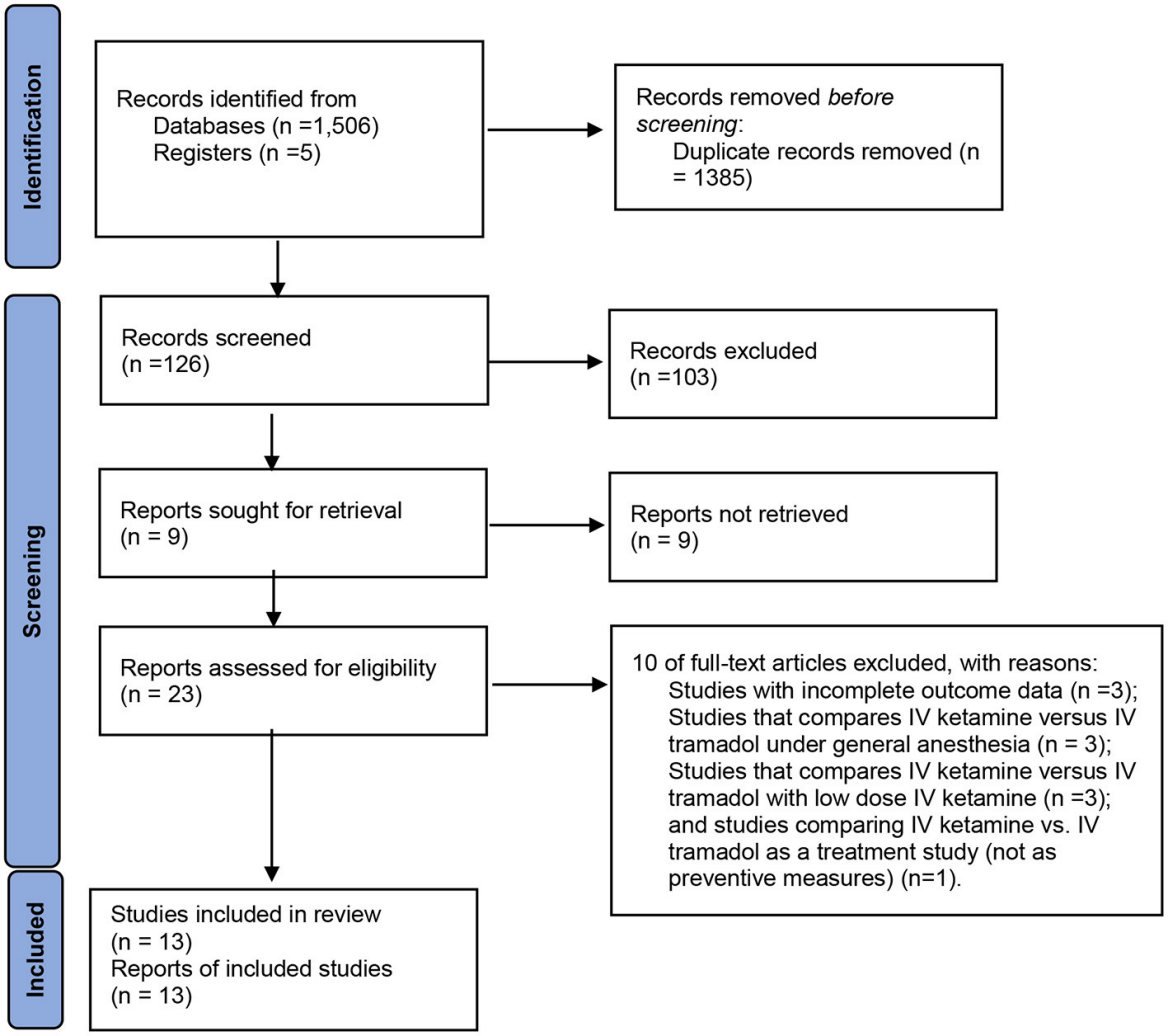
PRISMA 2020 flow diagram.

Figure 2 demonstrates the flow chart of this meta-analysis. Thirteen RCTs (13, 18, 19, 25–34) were included in this meta-analysis, having 1,532 patients (Table 1). Eight trials (13, 26, 28–31, 33, 34) compared ketamine with tramadol; three trials (18, 32) compared ketamine with tramadol and ondansetron, clonidine (19), pethidine (27), or dexmedetomidine (25).

**Table 1:** Characteristics of included studies.

**References**	**Study participants**	**Sample size tramadol/ Ketamine**	**Type of operations**	**Drugs used for spinal anesthesia**	**Dose, route of ketamine and tramadol**	**Outcomes**
Akram et al. (28)	ASA class I–II, Age between 18 and 50 years, of either sex	32/32	Lower abdominal surgeries	3 ml of 0.5% heavy Bupivacaine	Ketamine 0.05 mg/kg IV, and Tramadol 1 mg/kg IV	Shivering was observed 6 (18.75%) in Group-K and 15 (46.88%) in Group-T (*p*-value = 0.01).
Ameta et al. (25)	ASA class I-II, aged 21–60 years, of either sex	50/50	Lower abdominal or lower limb surgeries	2.8 mL of 0.5% heavy bupivacaine	Ketamine 0.5 mg/kg IV, and tramadol 0.5 mg/kg IV	Shivering was seen in Group K was 46%, and Group T was 50%. No hallucinations or nausea/vomiting in both groups. Bradycardia 4% in Group K, 12% in Group T.
Azam et al. (29)	ASA I and II status, 1 aged 18–40 years	200/200	Cesarean section	1.8 ml of 0.5% heavy Bupivacaine.	0.5 mg/kg Ketamine IV, and 2 mg/kg tramadol IV	Shivering was observed in 72 (36%) patients of Group-T and 39 (19.5%) from Group-K (P = 0.000).
Cahyadi et al. (26)	ASA I and II status, aged 18-64 years	30/30	Lower abdominal or lower limb surgeries	Not specified	Ketamine IV 0.25 mg/kg, and Tramadol IV 0.5 mg/kg	Shivering was seen in 17 (56.7%) patients in Group-T and 17 (56.7%) from ketamine group (*P* = 0.942). The mean (SD) onset of shivering in minutes were 26.44 (19.708) and 25.33 (13.425) in Group-K and Group-T groups, respectively with *p*-value of 0.839.
Gangopadhyay et al. (27)	ASA I and II status, aged between 18 and 55 years	30/30	Infra-umbilical surgeries	3 ml of 0.5% heavy Bupivacaine	Ketamine 0.5 mg/kg IV, and tramadol 1.0 mg/kg IV, or	Shivering was seen in 4 patients of Group-T and 2 cases from ketamine. Nausea and vomiting (24 vs. 1); Pruritis (3 vs. no cases) in Group-T vs. Group-K. No evidence of respiratory depression, bradycardia, hypotension in both groups.
Hidayah et al. (30)	ASA I and II status, aged 18–70 years	50/50	Lower abdominal or lower limb surgeries	12.5 mg of 0.5% hyperbaric bupivacaine and 25 mcg fentanyl	Ketamine 0.5 mg/kg IV, and tramadol 0.5 mg/kg IV	The incidence of shivering was 4 (8%) cases in Group K, 8 (16%) patients in Group T. Hallucination (2 cases vs. 0); Nystagmus (39 vs. 0); Nausea and vomiting (9 vs. 6) from Group-K and Group-T, respectively.
Ilyas et al. (31)	ASA I and II status, aged 18–60 years	46/46	Lower abdominal procedures	15 mg of 0.5 % heavy bupivacaine.	Ketamine 0.05 mg/kg IV, and tramadol 1 mg/kg IV	Shivering was observed in 5 (10%) patients in ketamine group and 11 (24%) patients in Tramadol group.
Jouryabi et al. (32)	ASA I and II status, aged 18–40 years	127/127	Cesarean Section	12.5 mg isobaric bupivacaine.	Ketamine 0.2 mg/kg IV, and tramadol 0.5 mg/kg IV	Shivering was witnessed in 68 (53.5%), and 26 (20.5%); Nausea &vomiting [25 (19.7) vs 63 (49.6)]; Hypotension [7 (5.51) vs 28 (22.04)]; Bradycardia [0 (0) vs 14 (11)]; Hallucination [9 (7.1) vs 0 (0)]; Nystagmus [13 (10.2) vs 0 (0)]; Headache [5 (3.9) vs 10 (7.9)] in Groups- K & T respectively.
Lakhe et al. (18)	ASA I and II status, aged 18-65 years	30/30	General surgeries, Orthopedics or Gynecologic procedures	15 mg of 0.5 % heavy bupivacaine.	Ketamine 0.25 mg/kg IV, and tramadol 0.5 mg/kg IV	Shivering was present in 3 (10%) and 3 (10%) in Group-K & Group-T. Onset of shivering (mean ± sd) in minutes were 18.33 ± 2.88 and 16.67 ± 10.41 in Group-K & Group-T, respectively. Nausea, vomiting and hypotension were absent in both groups.
Lema et al. (13)	ASA I and II status, aged 18–39 years	41/41	Cesarean section	2.5 mL of 0.5% isobaric Bupivacaine.	Ketamine 0.2 mg/kg IV, and tramadol 0.5 mg/kg IV	Shivering was witnessed in 41.5% and 53.7%; Time to shivering in minutes was 27.5 ± 37 and 25 ± 27.7; Hypotension in 5 (12.2%) vs. 4 (9.8%); sedation 2 (4.9%) vs. 7 (17.1%), Nausea and vomiting 7 (17.1%) vs. 5 (12.2%) in Group-T & Group-K respectively; and no patient developed bradycardia and hallucinations.
Nazir et al. (33)	ASA I and II status, aged 18–60 years	30/30	Lower limb Surgeries	3 ml of 0.5% heavy Bupivacaine.	Ketamine 0.5 mg/kg IV, and tramadol 0.5mg/kg IV	Shivering was present in 3 (10%) and 2 (6.7%) in Group-K & Group-T. None of the patients had episodes of oxygen desaturation or respiratory depression, hallucinations, tachycardia, hypotension or hypertension.
Seyam et al. (34)	ASA I and II status, aged 21–60 years	50/50	Not specified	2.8 mL of 0.5% (14 mg) heavy bupivacaine	Ketamine 0.2 mg/kg IV, and tramadol 0.5 mg/kg IV	Shivering was observed in 28 (56%) and 18 (36%); Time to shivering in minutes was 31.5 ± 11 vs. 29.5 ± 9; Hypotension 11 (22%) vs 9 (18%); Nausea & vomiting 17 (34%) vs 11 (22%); Sedation (Rmsay score ≤ 2) was 5 (10%) vs 17 (34%) in Group-T & Group-K respectively. None of the patients had bradycardia or hallucinations.
Wason et al. (19)	A total of 200 patients (50 cases in each group), ASA I and II status, aged 21–60 years	50/50	Lower abdominal or lower limb surgery	2.8 mL (14 mg) of 0.5% heavy bupivacaine	Ketamine 0.5 mg/kg IV, and tramadol 0.5 mg/kg IV	Shivering was present in 9 (18%) vs. 6 (12%); Hypotension 12% (6/50) vs. 12% (6/50); Bradycardia 1 (2%) vs. 2 (4%); and Nausea 0(0%) vs 2 (4%) patients in Group-K & Group-T respectively. Sedation score (grades 3 and 4) was significantly higher in the Group-K.

In six RCTs (18, 19, 25–27, 30), patients underwent lower abdominal and lower limb surgeries; patients in 2 trials (28, 31) underwent lower abdominal surgeries; patients in 3 trials (13, 29, 32) underwent cesarean section, and patients in a single trial (33) underwent lower limb surgery. However, a single trial (34) did not report the specific type of surgery.

Regarding the dose of bupivacaine used for spinal anesthesia, five trials (18, 27, 28, 31, 33) administered 15 mg of heavy bupivacaine, three studies (19, 25, 34) used 14 mg of heavy bupivacaine, and three studies (13, 30, 32) administered 12.5 mg of heavy bupivacaine, and a single trial (29) administered 9 mg of heavy bupivacaine. However, one trial (26) did not report the dose of local anesthetics used for spinal anesthesia.

### The effect of ketamine vs. tramadol on the prevention of shivering

**Figure 3:**
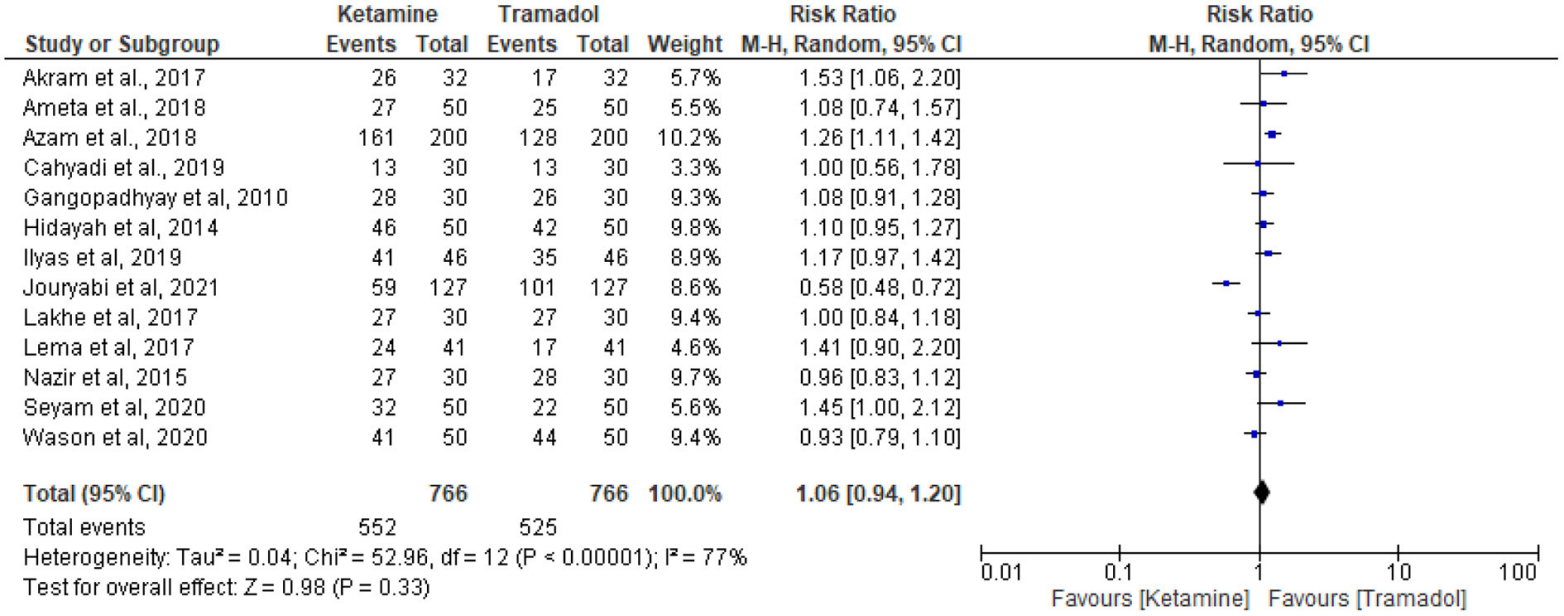
The effect of ketamine vs. tramadol on the prevention of shivering following spinal anesthesia.

**Figure 4:**
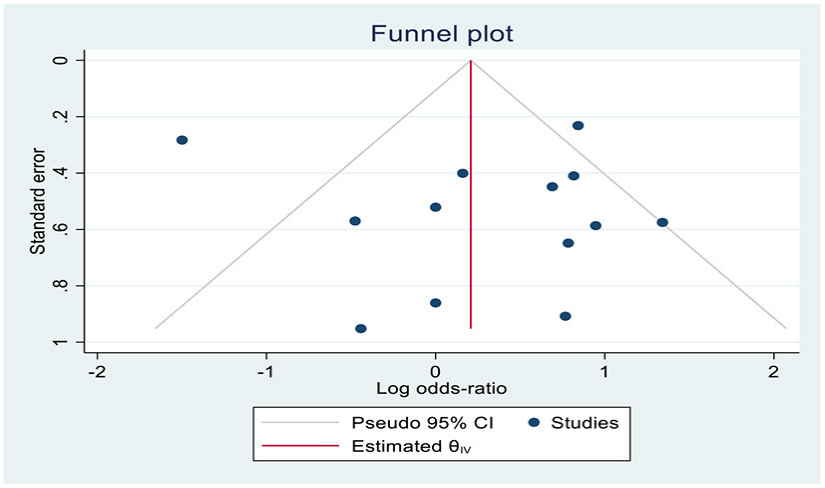
Funnel plot of the effect of tramadol vs. ketamine on prevention of shivering.

Thirteen RCTs (13, 18, 19, 25–34) reported the effective rate of shivering control. The random effects model was utilized because the value of *I*^2^ was >50%. The effective rate of shivering control was comparable between groups (RR =1.06; 95% CI [0.94, 1.20], *P* = 0.33, *I*^2^ = 77%) (Figure 3). Sensitivity analysis was executed for the effective rate of shivering control by excluding a single study consecutively but with no source of heterogeneity detected and publication bias detected (Figure 4).

### The effect of tramadol vs. ketamine on onset of shivering

**Figure 5:**
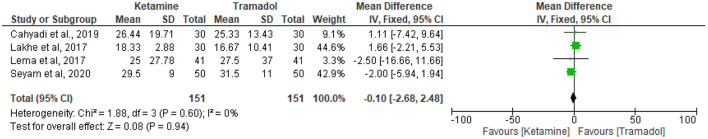
The effect of tramadol vs. ketamine on onset of shivering.

Four RCTs (13, 18, 26, 34) compared time to onset of shivering of ketamine vs. tramadol. Since there was no heterogeneity detected (*I*^2^ = 0%), the fixed effect model was utilized. The result showed that there was no significant differences regarding time to the onset of shivering time in minutes (MD = −0.10; 95%CI [– 2.68, 2.48], *P* = 0.94, *I*^2^ = 0%) (Figure 5).

### The effect of tramadol vs. ketamine on the incidence of nausea and vomiting

**Figure 6:**
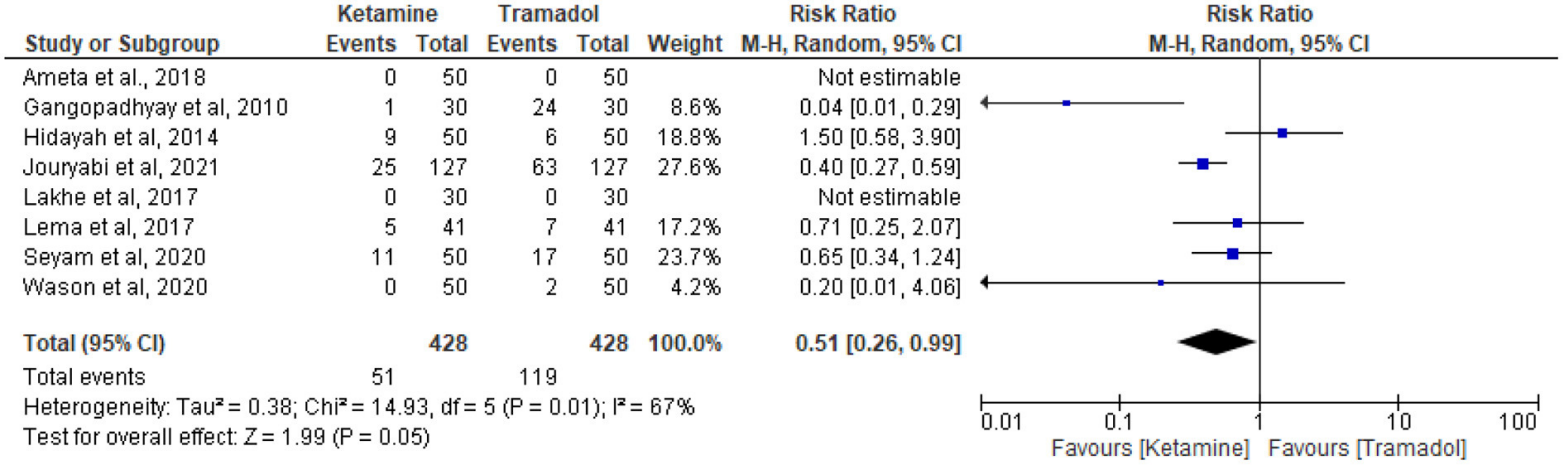
The effect of tramadol vs. ketamine on the occurrence of nausea and/or vomiting.

Eight articles reported the incidence of nausea and vomiting (13, 18, 19, 25, 27, 30, 32, 34). Fifty-one patients receiving intravenous ketamine and 119 patients receiving intravenous tramadol experienced nausea and vomiting out of 428 patients in each group. Ketamine had lower incidences of nausea and vomiting than tramadol (RR = 0.51; 95%CI [0.26, 0.99], P = 0.05, *I*^2^ = 67%) (Figure 6).

### The effect of tramadol vs. ketamine on the incidence of hypotension

**Figure 7:**
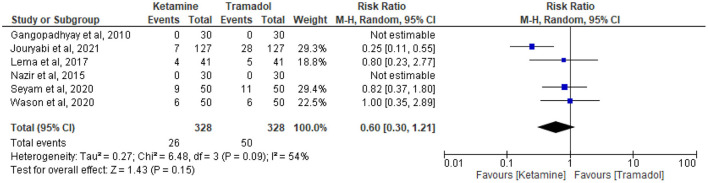
The effect of tramadol vs. ketamine on the incidence of hypotension.

The incidence of hypotension was reported in six trials (13, 19, 27, 32–34). Twenty-six patients receiving ketamine and 50 patients receiving tramadol experienced hypotension out of 328 patients in each group. Tramadol had comparable results with ketamine regarding the incidence of hypotension (RR = 0.60; 95%CI [0.30, 1.21], *P* = 0.15, *I*^2^ = 54%) (Figure 7).

### The effect of tramadol vs. ketamine on the incidence of bradycardia

**Figure 8:**
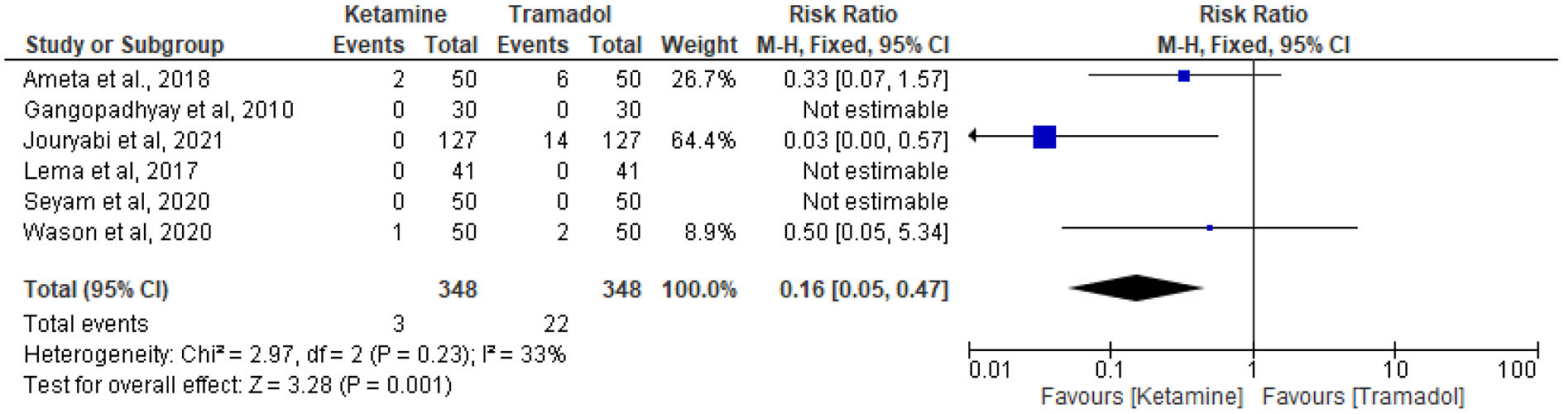
The effect of tramadol vs. ketamine on the incidence of bradycardia.

The incidence of bradycardia was reported in six trials (13, 19, 25, 27, 32, 34). Three patients receiving ketamine and 22 patients receiving tramadol experienced bradycardia out of 348 patients in each group. Tramadol was associated with higher incidence of bradycardia (RR = 0.16; 95%CI [0.05, 0.47], *P* = 0.001, *I*^2^ = 33%) (Figure 8).

### The effect of tramadol vs. ketamine on the incidence of hallucinations

**Figure 9:**
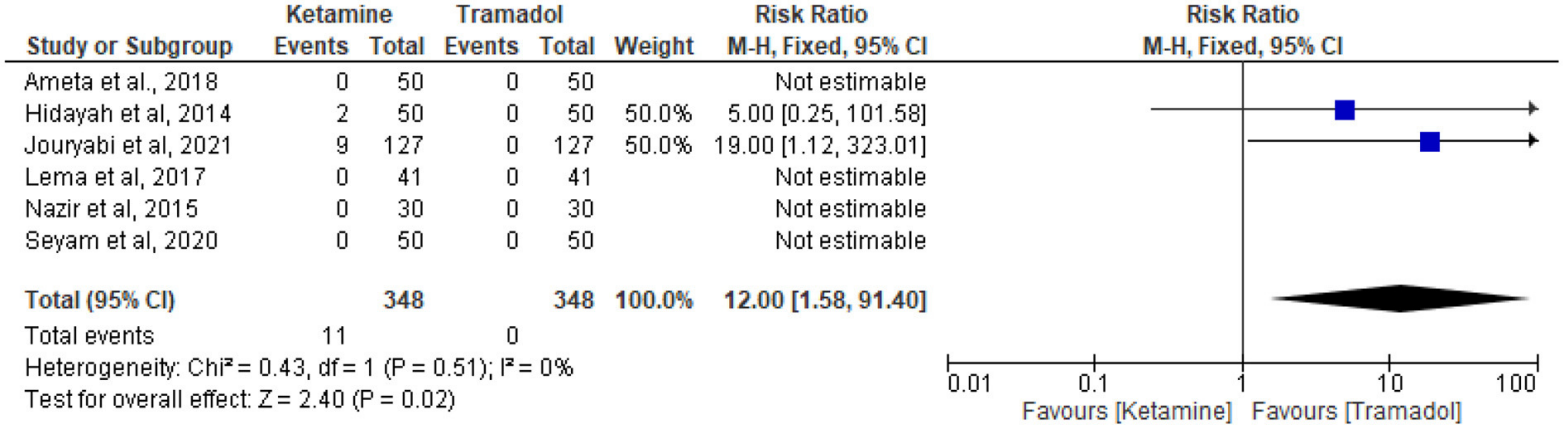
The effect of tramadol vs. ketamine on the incidence of hallucinations.

The incidence of hallucination was reported in six RCTs (13, 25, 30, 32–34). Eleven patients in the ketamine group and no patient in the tramadol group experienced hallucination in a total of 348 patients in each group. Ketamine was associated with higher incidence of hallucinations (RR = 12; 95%CI [1.58, 91.40], *P* = 0.02, *I*^2^ = 0%) (Figure 9).

## Discussion

Post-spinal anesthetic shivering is a common complication following subarachnoid administration of local anesthetics which results in repression of thermoregulatory mechanism for hypothermia (14, 34). In this meta-analysis, we compared the effect of intravenous ketamine and tramadol on the prevention of post-spinal anesthetics shivering.

In this meta-analysis, ketamine had comparable effects to tramadol in preventing post-spinal anesthetic shivering with a *P*-value of 0.51; and there were no significant differences regarding the onset of shivering with a *P*-value of 0.94. Tramadol inhibits the reuptake of serotonin and noradrenaline in the spinal cord and also has an effect on alpha-2 adrenergic and opioid receptors that might have anti-shivering effects (35–37). Ketamine is a competitive NMDA (N-Methyl D-Aspartate) receptor antagonist that inhibits noradrenergic and serotonergic neurons that might result in anti-shivering effects. Intravenous administration of ketamine and tramadol can be used for preventing post-spinal anesthetic shivering (19, 33, 34, 38).

The current study included a pooled analysis of the incidences of adverse events (nausea and vomiting, bradycardia, hypotension, and hallucinations) after the administration of anti-shivering agents. Ketamine showed a better outcome with less occurrences of nausea and vomiting (*P* = 0.03) and bradycardia (*P* = 0.001). Ketamine can cause a dose-dependent direct stimulation of the central nervous system that leads to an activation of the sympathetic nervous system and sustains heart rate (39). Ketamine was associated with a higher incidence of hallucinations (*P* = 0.02), and this might be due to its effect on glutamatergic signaling in psychosis that results in hallucination (40, 41). Intravenous ketamine had a comparable incidence of hypotension with intravenous tramadol with a *P*-value of 0.15. Research included in this meta-analysis (13, 19, 34) reported that ketamine was related with higher sedation scores than tramadol, despite the fact that we have not performed a pooled analysis due to variances in sedation scales employed in the included studies. Ketamine may be a more effective anti-shivering medicine in this context than tramadol because of the higher sedation scores in the ketamine group, which may be crucial in maintaining optimal surgical circumstances and decreasing patient pain following spinal anesthesia. However, because there are variances in the types and dosages of local anesthetics used for spinal anesthesia as well as the types and durations of the procedures carried out in the included RCTs, the findings may be highly heterogeneous.

The main limitation of this meta-analysis might be the relatively inadequate sample size to make generalizations, and therefore further studies should be conducted. The other limitation of this meta-analysis could be the heterogeneity of the scales used for shivering to run a pooled analysis.

## Conclusions

Intravenous ketamine and tramadol are comparable in the prevention of post-spinal anesthetic shivering. Ketamine could be a better anti-shivering agent with less occurrences of nausea, vomiting, and bradycardia. Ketamine had comparable effects regarding the incidence of hypotension. However, ketamine was associated with higher incidences of hallucinations in comparison to tramadol.

## Data availability statement

The original contributions presented in the study are included in the article/supplementary material, further inquiries can be directed to the corresponding author.

## Author contributions

EF, SK, MH, TT, and DT conceived the data, participated in the study design, conducted the statistical analysis, and drafted the manuscript. EF, TT, MH, and DT were involved in collecting the data, performing data analysis, and drafting the manuscript. YF and SS have also participated in the study design, data analysis, and revising of the manuscript. All authors have read and approved the manuscript.

## Conflict of interest

The authors declare that the research was conducted in the absence of any commercial or financial relationships that could be construed as a potential conflict of interest.

## Publisher's note

All claims expressed in this article are solely those of the authors and do not necessarily represent those of their affiliated organizations, or those of the publisher, the editors and the reviewers. Any product that may be evaluated in this article, or claim that may be made by its manufacturer, is not guaranteed or endorsed by the publisher.

## Abbreviations

MD: Mean difference
RR: Relative risk
CI: Confidence interval
RCTs: Randomized controlled trials.
